# Comprehensive Analysis of the Expression, Prognostic Value, and Immune Infiltration Activities of GABRD in Colon Adenocarcinoma

**DOI:** 10.1155/2023/8709458

**Published:** 2023-05-02

**Authors:** Fakun Huang, Zhengyang Wang, Liyue Zhu, Changqing Lin, Jia-xing Wang

**Affiliations:** ^1^Department of Gastrointestinal Surgery, The First Affiliated Hospital of Fujian Medical University, Fujian, China; ^2^Fujian Medical University, Fuzhou, Fujian, China

## Abstract

Colon adenocarcinoma (COAD) is one of the tumors with the highest mortality rates. It is of the utmost significance to make an accurate prognostic assessment and to tailor one's treatment to the specific needs of the patient. Multiple lines of evidence point to the possibility that genetic variables and clinicopathological traits are connected to the onset and development of cancer. In the past, a number of studies have revealed that gamma-aminobutyric acid type A receptor subunit delta (GABRD) plays a role in the advancement of a number of different cancers. However, its function in COAD was rarely reported. In this study, we analyzed TCGA datasets and identified 29 survival-related differentially expressed genes (DEGs) in COAD patients. In particular, GABRD expression was noticeably elevated in COAD specimens. There was a correlation between high GABRD expression and an advanced clinical stage. According to the results of the survival tests, patients whose GABRD expression was high had a lower overall survival time and progression-free survival time than those whose GABRD expression was low. GABRD expression was found to be an independent predictive predictor for overall survival, as determined by multivariate COX regression analysis. Additionally, the predictive nomogram model can accurately predict the fate of individuals with COAD. In addition, we observed that GABRD expressions were positively associated with the expression of T cells regulatory (Tregs), macrophages M0, while negatively associated with the expression of T cells CD8, T cells follicular helper, macrophages M1, dendritic cells activated, eosinophils, and T cells CD4 memory activated. The IC50 of BI-2536, bleomycin, embelin, FR-180204, GW843682X, LY317615, NSC-207895, rTRAIL, and VX-11e was higher in the GABRD high-expression group. In conclusion, we have shown evidence that GABRD is a novel biomarker that is connected with immune cell infiltration in COAD and may be utilized to predict the prognosis of COAD patients.

## 1. Introduction

Colon adenocarcinoma (COAD) ranks third and fourth place in the rankings of cancer incidence and mortality all over the world, respectively [1]. It is well known that variables such as dietary choices, age, obesity, smoking, and a lack of physical activity are risk factors for COAD [2, 3]. The prevalence of COAD varies greatly from nation to nation. It is believed that a number of different causes are responsible for this variation in occurrence [4, 5]. To be more specific, among other things, socioeconomic status is important, with a poor socioeconomic level being related to an increased risk of developing COAD [6, 7]. The death rate associated with COAD has declined by around 35% from 1990 to 2007, and it is presently down approximately 50% from its highest mortality rate. This decline can be attributed to effective screening techniques, early interventions, and improved treatment choices. However, it is important to highlight that the decrease in overall mortality from COAD may have obscured the death rate for young adult patients with COAD [8, 9]. Despite the fact that target therapies, chemotherapy, and surgery have considerably improved the overall survival of COAD patients, around half of all COAD patients will eventually develop distant metastases, which is also the most common reason why treatments do not work [10, 11]. If the tumor has metastasized to other organs, the 5-year mortality rate declines drastically to 8.1%. Biomarkers, such as the microsatellite instability state, the BRAF mutation state, and the RAS mutation state, have been utilized to assist in the identification of patients who are at an increased risk of the progression or recurrence of their tumors [12, 13]. As a consequence of this, one of the primary focuses of COAD research has shifted to the identification of molecular abnormalities in COAD patients.

The process of tumorigenesis is intimately connected to the properties of cancer cells in and of themselves, and it is an essential component of the immune system [14, 15]. Immune cells serve a vital function in immune surveillance and are critical components of the tumor microenvironment (TME) [16]. Tumor-infiltrating lymphocytes (TILs), myeloid-derived suppressor cells (MDSCs), tumor-associated macrophages (TAMs), and regulatory T cells (Tregs) are all components of the immunosuppressive microenvironment that have recently been shown to predict poorer outcomes in solid tumors like melanoma, breast, lung, ovarian, bladder, prostate, and renal cancer [17, 18]. Immunotherapy is currently considered a typical component of treatment for a variety of solid tumors. This is due to the fact that the immune system shows a different status in tumor patients and is inexorably related to the formation of tumors. High levels of stromal cells and immune cell infiltration are present in COAD at an early stage. Monoclonal antibodies, checkpoint inhibitors, therapeutic vaccines, adoptive cell therapy, adjuvant immunotherapy, and cytokines and oncolytic virus treatments are the six groups that make up anticancer immunotherapeutic methods [19, 20]. However, the inadequate immune response has been a problem for a long time, particularly for checkpoint inhibitors targeting PD-1 and PD-L1s in COAD [21, 22]. This is especially true in COAD. In particular, the use of ICIs, which have been shown to have little to no therapeutic efficacy in the majority of patients with metastatic COAD, in view of the fact that there are presently no drugs that have been proven to be successful and the fact that COAD is related to low rates of survival, immunosuppressive mechanisms that occur inside the tumor microenvironment may offer intriguing targets for future immunotherapy [23, 24]. This is especially relevant when considering the context of the current situation. Therefore, defining the immunophenotype of tumor-immune interactions and finding novel indicators and therapeutic options for COAD are both essential.

Gamma-aminobutyric acid type A receptor subunit delta (GABRD) is a ligand-gated ion channel-type receptor that has been linked to a wide range of neurological and psychiatric disease-related symptoms as well as the progression of cancer [25]. Recent research has shed light on the potential functional functions that GABRD played in the development of malignancies. For instance, Zhang et al. reported that patients who have IDH WT low-grade glioma and have GABRD expression on their tumors could benefit from its use as a possible independent prognostic marker. During this time, its expression was shown to have a negative correlation with the degree of TIM, which may assist to explain the beneficial conclusion of the survival analysis. It is possible that Cg13916816 is an important CpG site that influences GABRD expression in IDH wild-type low-grade gliomas [26]. Sawaki et al. reported that having a high level of GABRD mRNA expression in primary human gastric cancer tissue was related to a poor prognosis. In comparison to the expression of control siRNA, the expression of siGABRD in gastric cancer cells resulted in a considerable reduction of cellular proliferation and invasion, as well as an increase in apoptosis. The growth of gastric cancer cells was suppressed in vitro by anti-GABRD polyclonal antibodies, which also led to a reduction in the size of peritoneal tumor nodules in the mouse xenograft model. It has been suggested that GABRD may be a viable therapeutic target for gastric cancer since its expression is increased in gastric cancer tissue and it is related to a poor prognosis [27]. In colorectal cancer, GABRD has been hypothesized to have a role similar to that of a tumor promoter in a previous study [28]. On the other hand, the clinical relevance of GABRD and its connection with TME were only infrequently recorded.

## 2. Materials and Methods

### 2.1. Patient Samples

The TCGA website was accessed in order to retrieve the mRNA expression data as well as the pertinent clinical information for COAD patients. It is now being gathered that there are 41 normal tissues and 480 COAD tissues. Patients from the TCGA who were included in later research but lacked necessary clinical information were excluded. The following clinicopathological features of patients were recorded: age, gender, and stage.

### 2.2. Pan-Cancer Analysis

The ONCOMINE database is an integrated online data-mining platform that offers a comprehensive examination of the expression of the genome across various tumor samples as well as normal control samples. In the course of our research, we compared the levels of GABRD transcription found in COAD samples to those found in normal neighboring tissues. The level of statistical significance was determined to be attained when *p* was less than 0.05, the fold change (FC) was fixed at 2, and the cutoff for statistical significance was established at 10%.

### 2.3. Differential Expression Analysis

In order to examine the gene expression matrix for differences between COAD samples and normal colon samples, the limma software was utilized. |log 2(FC)| > 2 and a false discovery rate (FDR) of less than 0.05 were required in order to classify genes as differentially expressed genes (DEGs).

### 2.4. Survival Assays according to GABRD Expressions in COAD

After classifying TCGA-COAD patients into high-expression and low-expression groups according to the levels of each GABRD, the differences were analyzed using the Kaplan-Meier methods, taking into consideration the survival information revealed by the “Surv_cutpoint” function in the survminer R package [29]. An investigation into whether or not there was a link exists between the OS features of COAD patients and the expressions of each GABRD was carried out by employing the “survival” package to conduct univariate assays. In order to determine whether or not these variables may be considered independent predictors of OS, multivariate assays were carried out with the “survival” package.

### 2.5. Functional Enrichment Analysis

Patients diagnosed with COAD who participated in the TCGA were classified into high and low GABRD expression groups, respectively, based on the median expression of GABRD expression. The DEG analysis between these two groups was carried out with the help of the R software DESeq2, and the criterion for DEGs was determined to be an adjusted *p* value of less than 0.05 and a log2-fold-change (FC) of more than 1. Spearman's correlation analysis was utilized to examine the degree of overlap that exists between the expression of the top 10 DEGs and GABRD. The “GOplot R” program was used to carry out functional enrichment studies on the DEGs. These analyses included Gene Ontology (GO) and Kyoto Encyclopedia of Genes and Genomes (KEGG) analyses. The GSEA was performed with the R package clusterProfiler, and statistically substantially enriched function or pathway words were considered to have an adjusted *p* value of less than 0.05 and a false discovery rate of less than 0.25 [30].

### 2.6. Nomogram Construction

Combining the findings of the genetic risk score model with clinical characteristics led to the development of a nomogram that was able to accurately forecast the 3- and 5-year OS of COAD. The calibration plot was used to evaluate the nomogram's ability to make accurate predictions. The area under the curve (AUC) was used to analyze the time-dependent sensitivities and specificities of the nomogram for both the 3-year and 5-year OS ROC curves. R was the statistical program of choice for all of the statistical studies that were conducted. The rms package was used to create the nomogram and calibration plots, and the timeROC package was used to conduct the analysis of the time-dependent ROC curve. Both packages are part of the R program. The Hmisc package of the R program was used to conduct comparisons of the C-index between the nomogram and the staging systems developed by the American Joint Committee on Cancer. If the *p* values were lower than 0.05, then the null hypothesis that there was no difference was rejected.

### 2.7. Analysis of the Relative Proportions of Tumor-Infiltrating Immune Cells (TIICs) in COAD Tissues

The CIBERSORT deconvolution technique was utilized in order to evaluate the TIICs present in COAD samples that were taken from the TCGA cohort [31]. Using the CIBERSORT platform, we were able to derive the gene expression signature matrix, consisting of 22 TIICs. The matrix data of gene expression levels were compared with those of the signature matrix of 22 TIICs from the CIBERSORT platform. This *p* value serves as a measure of confidence in the data that was collected. Inferred proportions of TIICs were evaluated by CIBERSORT, and when the criterion of *p* < 0.05 was met, the findings of those evaluations were deemed to be accurate. Because of this, the only samples that were considered eligible for further analysis were those with a CIBERSORT *p* value of less than 0.05. In addition to that, the default setting for the signature matrix's number of permutations was set to 100.

### 2.8. Immunoassay

We analyzed the link between GABRD expression and TILs by using the data from TIMER and TCGA. This allowed us to evaluate whether or not there was a connection between GABRD and TILs. In order to further investigate the impact that GABRD has on TILs, the interaction between GABRD and immunological checkpoints in each of the three groups was investigated. The tumor immune dysfunction and exclusion (TIDE) method was used to provide a prediction on the potential ICI response.

### 2.9. Drug Sensitivity Prediction

For the purpose of predicting the IC50 of chemotherapeutic medications, the “pRRophetic” R package was utilized. The IC50 is a figure that reflects how efficient a substance is at blocking particular biological or biochemical processes.

### 2.10. Statistical Analysis

Statistical analyses were performed using R software v3.5.0 (R Foundation for Statistical Computing, Vienna, Austria) and GraphPad Prism v7.00 (GraphPad Software Inc., USA). The data were put through several statistical tests that are considered to be conventional. The FDR approach was used to make adjustments for the multiple testing. *p* values were two-sided, and a value of less than 0.05 was regarded as statistically significant.

## 3. Results

### 3.1. Identification of the Survival-Related DEGs in COAD Patients

**Figure 1:**
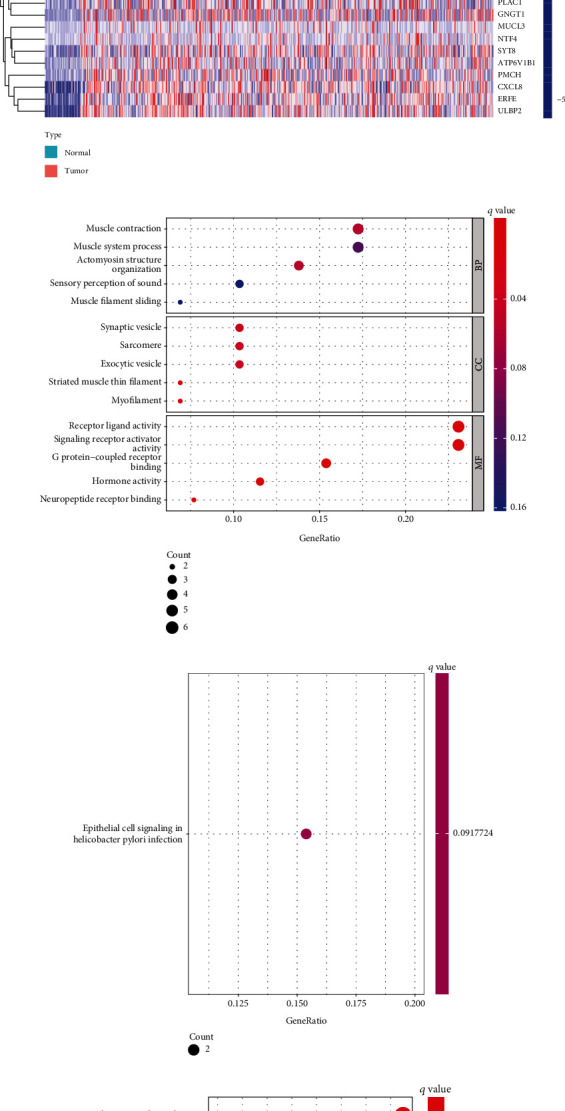
Identification of the survival-related DEGs in COAD patients. (a) Volcano plot of 2088 DEGs in COAD specimens, including 1000 downregulated genes and 1088 upregulated genes. (b) Venn diagram showed 29 survival-related DEGs in COAD. (c) The expressing pattern of 29 survival-related DEGs in COAD shown in heat map. (d) Bubble graph for GO enrichments. (e) Barplot graph for KEGG pathways. (f) Disease ontology enrichment analysis of the 29 survival-related DEGs in COAD.

Firstly, we compared the DEGs of COAD specimens and nontumor specimens against one another. As can be seen in Figure 1(a), we identified 2088 DEGs in COAD specimens, comprising 1000 genes with a downregulation and 1088 genes with an upregulation. After that, we carried out survival tests and located 309 genes associated with the process of survival. A Venn diagram was used to display the genes that overlapped one another (Figure 1(b)). The heat map displayed the expression pattern of 29 genes that overlapped one another (Figure 1(c)). To further explore the potential function of the critical 29 genes, we performed GO analysis, and the results indicated that 29 genes were mainly enriched in muscle contraction, muscle system process, actomyosin structure organization, synaptic vesicle, sarcomere, exocytic vesicle, receptor-ligand activity, signaling receptor activator activity, and G protein-coupled receptor binding (Figure 1(d)). KEGG assays suggested that 29 genes were mainly associated with epithelial cell signaling in *Helicobacter pylori* infection (Figure 1(e)). Moreover, we performed DO assays and found that 29 genes were mainly associated with obstructive lung disease, lung disease, preeclampsia, allergic rhinitis, and nasal cavity disease (Figure 1(f)).

### 3.2. GABRD Expression in COAD and Normal Tissues

**Figure 2:**
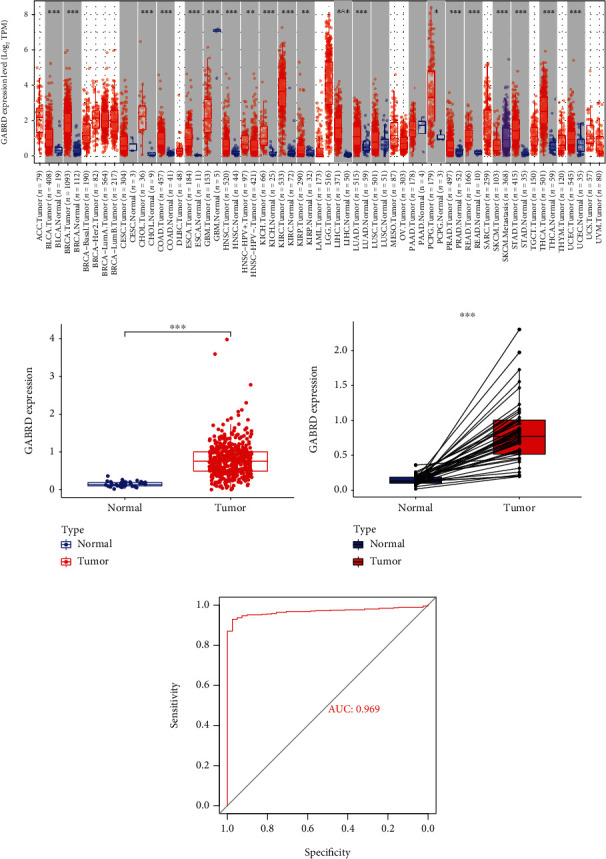
GABRD was highly expressed in COAD patients. (a) Pan-cancer assays of GABRD expression based on TCGA datasets. (b, c) A high expression of GABRD was observed in COAD specimens compared with nontumor specimens. (d) The diagnostic values of GABRD expression were confirmed in screening COAD specimens from normal specimens based on TCGA datasets. ^∗∗^*p* < 0.01, ^∗∗∗^*p* < 0.001.

Then, we used those 29 genes to do a search in the database known as “PubMed,” and we discovered that several of those genes had been investigated in the context of a variety of malignancies, including COAD. On the other hand, very little information on the expression and function of GABRD in COAD has been documented. As a result, we concentrated on GABRD. To begin, we carried out pan-cancer tests and discovered that GABRD displayed a dysregulated level in a wide variety of cancers. This led us to hypothesize that it may play a role in the growth of malignancies as a regulator (Figure 2(a)). Importantly, we showed that GABRD expression was markedly elevated in COAD tissues compared with nontumor specimens (Figures 2(b) and 2(c)). The purpose of this study was to investigate the diagnostic utility of GABRD expression in COAD patients. To do so, we carried out an ROC curve analysis, which revealed that GABRD was a potential indicator for distinguishing COAD specimens from nontumor specimens, with an area under the curve (AUC) of 0.969 (Figure 2(d)).

### 3.3. The Prognostic Value of GABRD Expression in COAD Patients

**Figure 3:**
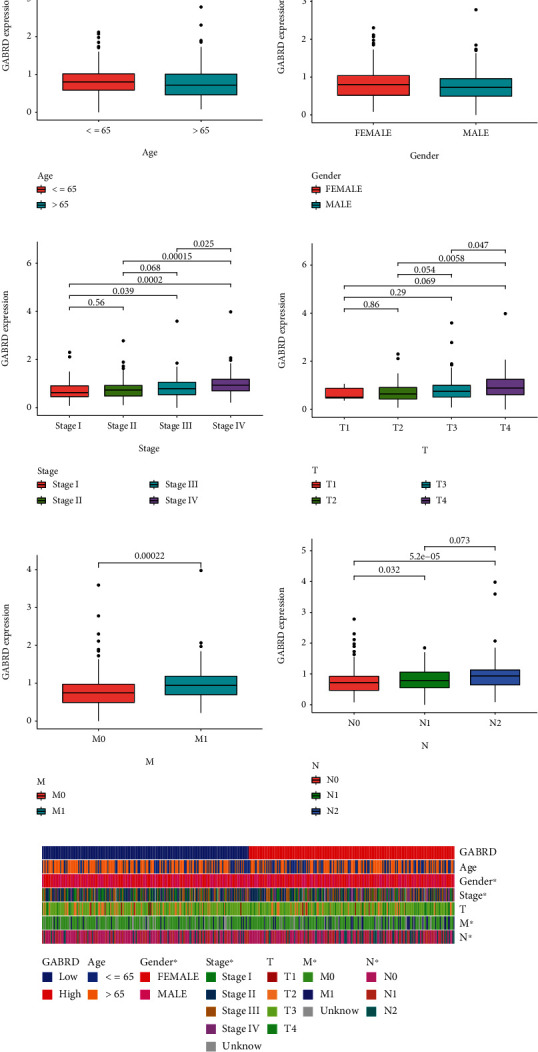
Association between the expression of GABRD and clinical characteristics in COAD patients. (a) Age, (b) gender, (c) clinical stage, (d) T stage, (e) M stage, (f) N stage. (g) The association between GABRD expression and different clinical factors was shown using heat map. ^∗^*p* < 0.05.

**Figure 4:**
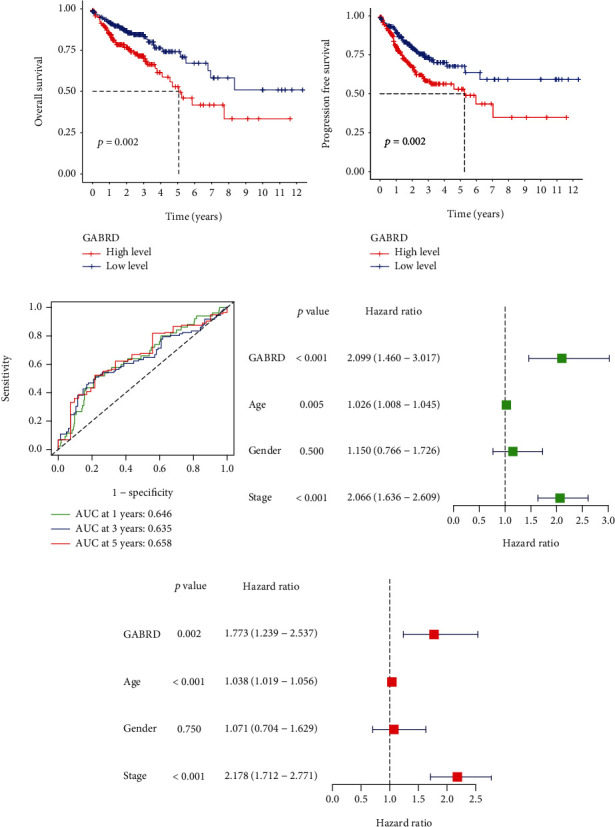
Correlation between GABRD expression and the long-term survival of COAD patients. (a, b) The Kaplan-Meier survival curves between groups with high GABRD expression and groups with low GABRD expression. (c) ROC curves drawn using TCGA data. (d, e) Univariate Cox analysis and multivariate Cox regression analysis of the risk factors related to the survival of COAD.

**Figure 5:**
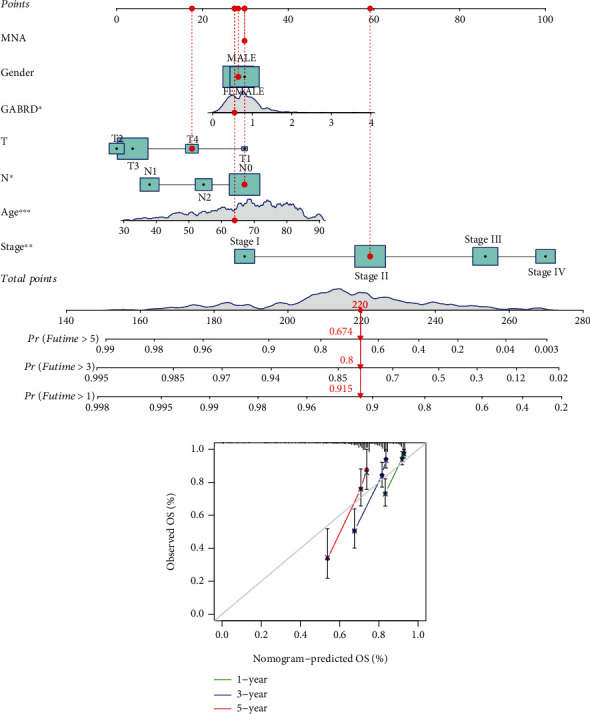
Validation of the nomogram for determining the overall survivals of COAD patients using data from the TCGA. (a) A prognostic nomogram for COAD patients that predicts their overall survival rate at one, two, and three years. (b) The ideal nomogram is represented by the dashed diagonal line on the calibration curve for the OS nomogram model. ^∗^*p* < 0.05, ^∗∗^*p* < 0.01, ^∗∗∗^*p* < 0.001.

To determine the relevance of GABRD expression to clinical practice, we analyzed associations between GABRD levels and other clinical factors in COAD patients. We discovered that COAD patients above the age of 65 had significantly higher levels of GABRD expression than COAD patients under the age of 65 (Figure 3(a)). On the other hand, we did not find any discernible differences in the GABRD expression of male patients compared to female patients (Figure 3(b)). Importantly, we identified a correlation between high GABRD expression and advanced clinical stage in COAD patients (Figures 3(c)–3(f)). The use of a heat map was utilized in order to demonstrate the relationship between GABRD expression and several clinical variables (Figure 3(g)). The prognostic value of GABRD expression in COAD patients was investigated further, and the results of the Kaplan-Meier survival curves indicated that patients with high GABRD expression showed poorer overall survival (Figure 4(a)*p* = 0.002) and progression-free survival (Figure 4(b)*p* = 0.002) than patients with low GABRD expression. According to the findings of TCGA, the area under the curve (AUC) for GABRD expression was 0.658, which indicated that GABRD has a significant predictive potential for the survival of COAD patients (Figure 4(c)). According to the findings of a univariate Cox regression analysis, the overall survival rate was significantly affected by age (*p* = 0.005), stage (*p* < 0.001), and GABRD expression (*p* < 0.001) (Figure 4(d)). Additionally, the multivariate COX regression analysis demonstrated that GABRD expression was an independent predictive predictor for overall survival (Figure 4(e)). A quantitative strategy for predicting the chance of overall survival at 1, 3, and 5 years for COAD patients is provided by the expression level of GABRD, which is an independent prognostic risk factor (Figures 5(a) and 5(b)). Our findings suggested GABRD as a novel prognostic biomarker for COAD patients.

### 3.4. The Biological Functions of GABRD in COAD

**Figure 6:**
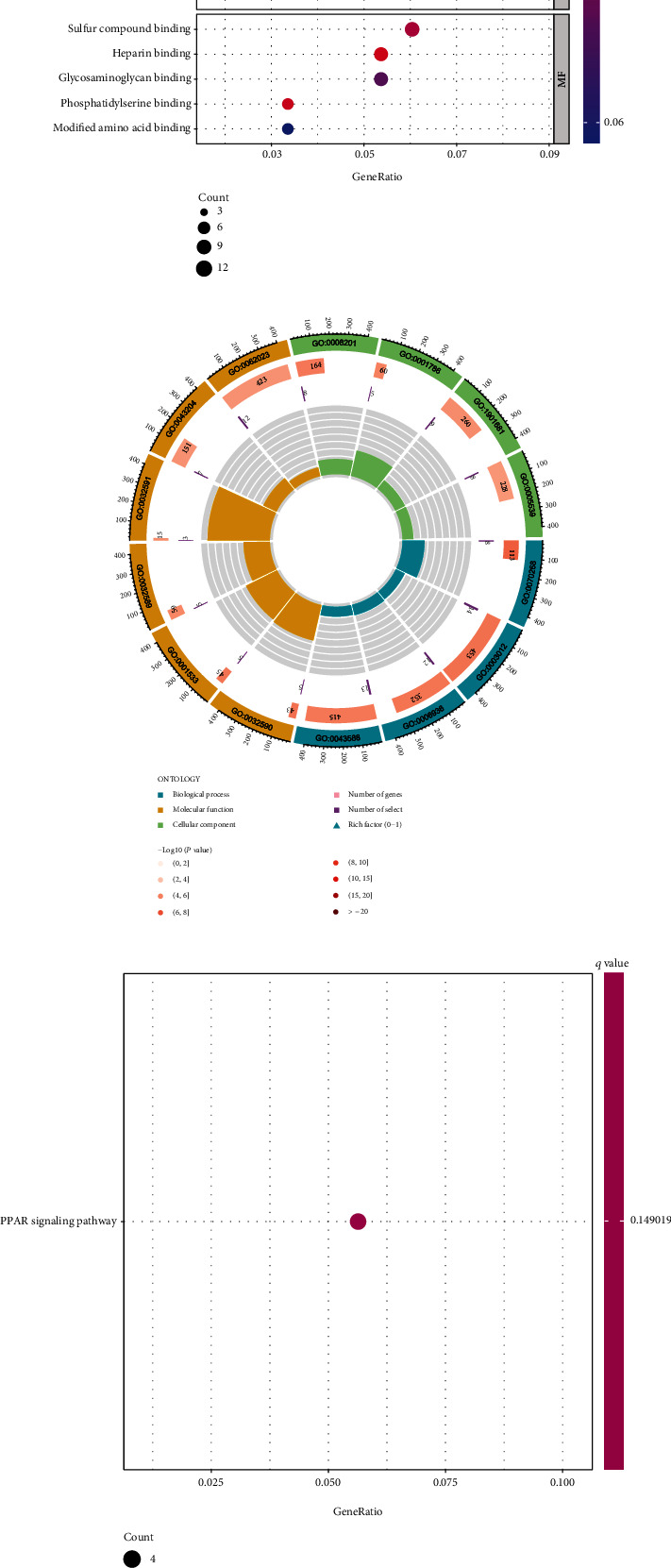
The function assays of GABRD-associated DEGs in COAD. (a) The representative DEGs associated with GABRD were shown in heat map. (b, c) Bubble graph for GO enrichment. (d) Barplot graph for KEGG pathways. (e) Gene set enrichment analysis.

A total of 236 DEGs were screened (Figure 6(a)). Functional annotation was conducted. GO assays revealed that GABRD-associated DEGs were mainly involved in the muscle system process, skin development, muscle contraction, perikaryon, dendrite membrane, cornified envelope, heparin binding, glycosaminoglycan binding, and sulfur compound binding (Figures 6(b) and 6(c)). Meanwhile, KEGG pathway analysis showed that GABRD-associated DEGs were mainly involved in the PPAR signaling pathway (Figure 6(d)). Since the level of GABRD expression was shown to be connected with the grade of the tumor and the prognosis of COAD patients, we formed the hypothesis that an increased level of GABRD expression accelerates the growth of tumors. Our group carried out GSEA and found that hallmarks of tumors such as ACUTE_MYELOID_LEUKEMIA, GLYCOSAMINOGLYCAN_BIOSYNTHESIS_CHONDROITIN_SULFAT, and _MTOR_SIGNALING_PATHWAY, NOTCH_SIGNALING_PATHWAY were dynamically correlated with the high GABRD expression, while OLFACTORY_TRANSDUCTION was significantly enriched in the low GABRD expression group (Figure 6(e)).

### 3.5. Distribution of Tumor-Infiltrating Immune Cells

**Figure 7:**
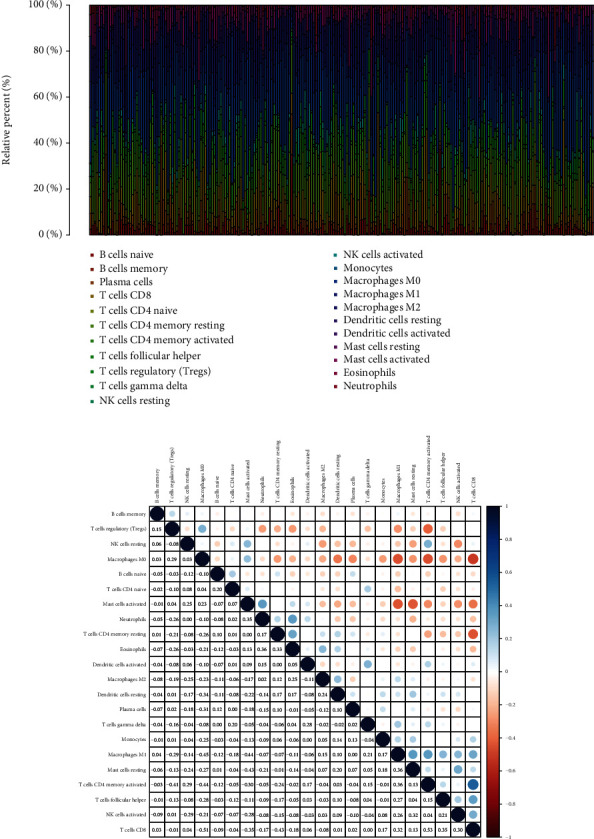
TIC profiles in tumor samples and correlation analysis. (a) A barplot illustrating the percentage of 21 different TIC types found in COAD tumor samples The names of the plot's columns were sample IDs. (b) A heat map displaying the association between 22 different types of TICs, with a number in each very small box representing the level of statistical significance of the link between the two types of cells.

**Figure 8:**
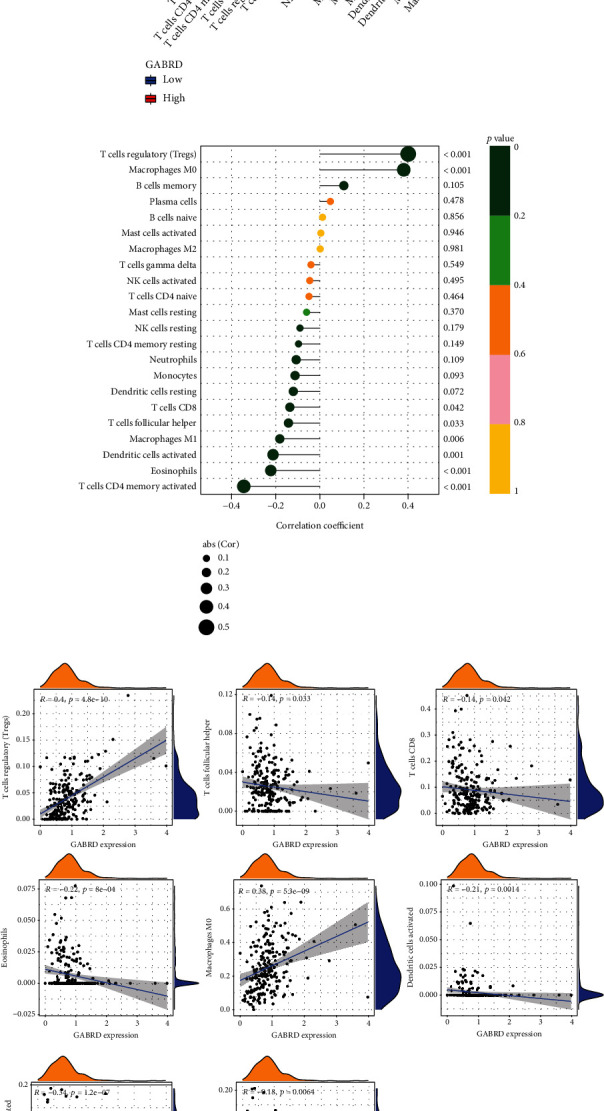
Correlation of TIC proportion with GABRD expression. (a) The ratio differentiation of 22 distinct types of immune cells was shown using a violin plot, and it was compared to the median level of BTK expression. This was done using COAD tumor samples with low or high GABRD expression levels. (b, c) Correlation between GABRD and infiltrating immune cells in COAD. ^∗^*p* < 0.05, ^∗∗^*p* < 0.01, ^∗∗∗^*p* < 0.001.

Through the application of the CIBERSORT approach, we investigated the pattern of immune cells. Figures 7(a) and 7(b), respectively, showed the makeup of it on COAD samples as well as the relationships among immune cells. A number of studies have demonstrated that immune cells have the potential to act as independent markers of survival rates and the efficacy of immunotherapy in COAD. The next step was to determine definitively whether or not the actions of GABRD were linked to immune cells. Importantly, we observed that GABRD showed a dysregulated level in several immune cells, including T cells regulatory (Tregs), T cells follicular helper, T cells CD4 memory activated, T cells CD8, macrophages M0, dendritic cells activated, mast cells resting, and eosinophils (Figure 8(a)). In addition, our group showed that GABRD expressions were positively related to the expressions of T cells regulatory (Tregs) and macrophages M0, while negatively associated with the expression of T cells CD8, T cells follicular helper, macrophages M1, dendritic cells activated, eosinophils, and T cells CD4 memory activated (Figures 8(b) and 8(c)).

### 3.6. Relationship between GABRD Expression and Immune Checkpoints in COAD

**Figure 9:**
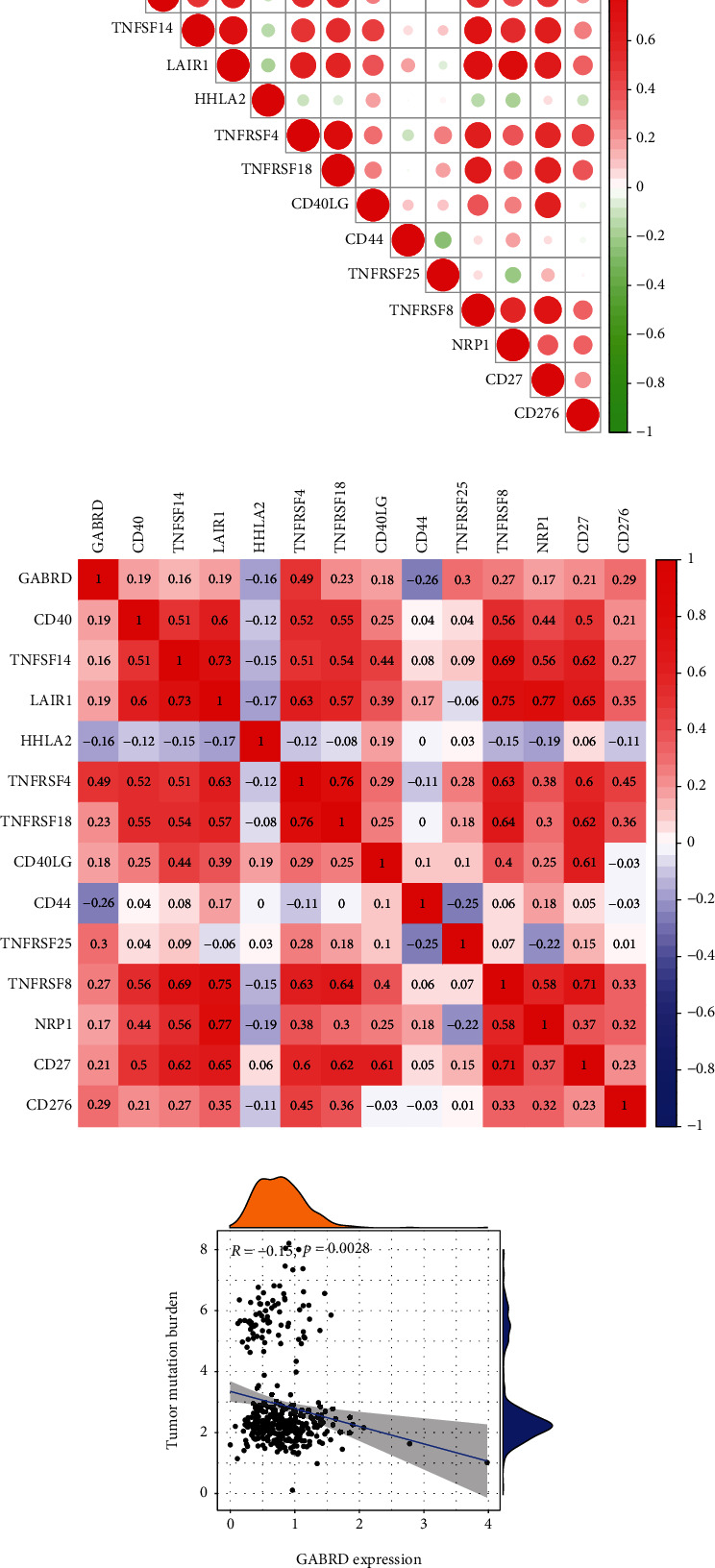
(a, b) The association between GABRD expression and immune checkpoints in COAD patients. (c) GABRD expression was negatively associated with tumor mutation burden.

Immune checkpoint genes (ICGs) are fundamental to the field of immunotherapy and have a role in both the onset and development of COAD. ICGs were shown to have a connection to both the beginning and the development of cancer by researchers. Additionally, it has been suggested that the ICGs have the potential to be therapeutic targets for ICB treatment. The examination of the clinical information and expression data on the many combinations of ICGs that are now accessible can be of assistance in finding targets for individualized treatment and in improving the efficacy of the present therapeutic approaches. Then, we found that GABRD expression was positively associated with CD40, TNFSF14, LAIR1, TNFRSF4, TNFRSF18, CD40LG, TNFRSF25, TNFRSF8, NRP1, CD27, and CD276 while negatively associated with HHLA2 and CD44 (Figures 9(a) and 9(b)). The tumor mutation burden (TMB) refers to the total number of somatic gene variations found per million bases of genomic DNA. These variants might be base substitutions, insertions, or deletions. At the genetic level, tumor cells are capable of producing a large number of specific mutations. Every 150 nonsynonymous mutations have the potential to produce one to two neoantigens. These neoantigens are able to be recognized by the autoimmune system, which in turn activates T cells and causes an immune response. We also found that GABRD expression was negatively associated with TMB (Figure 9(c)).

### 3.7. IC50 Score

**Figure 10:**
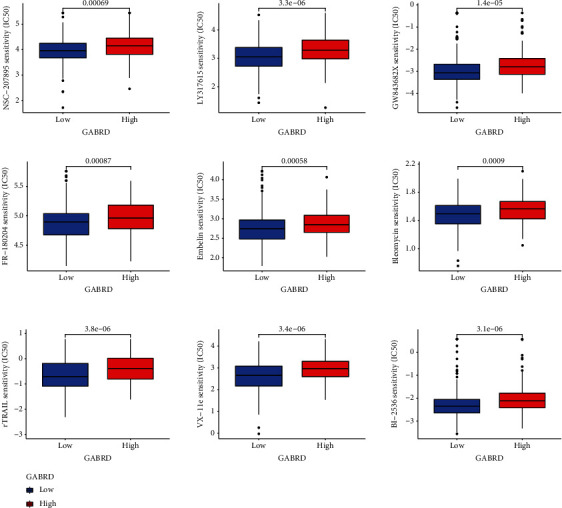
A breakdown of the IC50 values for several targeted medicines across the various GABRD expression groups.

When trying to assess how well patients may react to targeted pharmacological therapy, the IC50 is an important statistic that has to be employed. We were able to estimate probable alterations in the IC50 values of chemotherapeutic medications across the various GABRD expression groups by making use of the information that was supplied by the GDSC. The IC50 of BI-2536, bleomycin, embelin, FR-180204, GW843682X, LY317615, NSC-207895, rTRAIL, and VX-11e was higher in the GABRD high-expression group (Figure 10). As a consequence of this, the results showed that there was a significant distinction between the various GABRD expression groups in terms of the distribution of IC50 values for drugs that are specifically targeted.

### 3.8. Identification of GABRD Protein Expression in COAD Specimens

**Figure 11:**
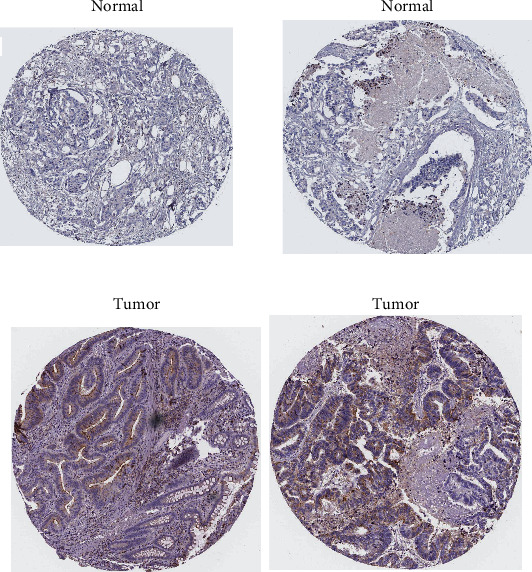
Images from the Human Protein Atlas, showing representative examples of immunohistochemistry staining for GABRD in normal tissues as well as COAD tissues: (a) normal tissues; (b) malignant tissues.

In addition, in order to evaluate the expression of GABRD in terms of the protein level, we requested immunohistochemistry pictures from the HPA database. It is obvious that the protein expression of COAD was significantly higher in tumor specimens than in normal specimens (Figures 11(a) and 11(b)).

## 4. Discussion

In recent years, COAD has emerged as an increasingly serious hazard to human health all over the world and has imposed a significant burden on society [32]. Even with recent advancements in surgery, radiation, chemotherapy, and immunotherapy, COAD still has a high propensity to spread and a dismal overall survival rate [33, 34]. In addition, the traditional TNM classification method determines a patient's stage of cancer based not on the patient themselves but rather on the location and size of the tumor [35, 36]. It is challenging to individually identify the result of a patient's condition. Therefore, discovering molecular prognostic indicators to estimate the risks and prognoses of individuals with COAD is vital for the purpose of directing treatment.

The development of throughput sequencing technology has helped shed light on illness-causing genes, expanded our knowledge of disease etiology, led to the discovery of new biomarkers, and fundamentally altered our perspective on the variety of human life [37, 38]. Researchers have identified a large number of genes connected to tumors that, when combined with genomic information, provide an accurate prediction of whether or not a patient's prognosis risk is high [39, 40]. By mining data on gene expression, a number of researchers have examined and evaluated numerous biomarkers connected to COAD [41, 42]. In this study, we performed a comprehensive analysis and identified 29 survival-related DEGs. Among them, our attention focused on GABRD, which was highly expressed in many types of tumors, including COAD. In addition, we also confirmed its diagnostic value in screening COAD specimens from nontumor specimens. Then, we analyzed its clinical significance and found that high GABRD expression was associated with an advanced clinical stage and a poor prognosis. More importantly, multivariate COX regression analysis confirmed that GABRD expression was an independent prognostic factor for overall survival. Our findings suggested GABRD as a novel biomarker for COAD patients.

The TME is made up of cancer cells, stromal cells, immune cells, and extracellular matrix, all of which have a substantial impact on the progression of cancer [43]. The TME contains cancer cells that can invade surrounding tissues either directly or indirectly through blood and lymphatic vessels [44]. These infiltrated cells have the ability to stimulate an immune response by cytokine receptors, releasing cytokines and other elements that influence the progression of the tumor. In recent years, fresh research has demonstrated that TME considerably alters the course of tumors and has shown a possible prognostic value for cancer prognosis, including COAD [45, 46]. These findings are now available to the general public. The fast growth of precision medicine has led to an increase in the number of studies in which researchers use statistical algorithms to investigate novel diagnostic and therapeutic targets. This homeostatic system contributes to the progression and recurrence of cancer, and it has significant ramifications for chemoresistant disease and immunotherapy. In addition to this, the therapeutic response is also affected by the nonimmune cellular components of the TME [47, 48]. For instance, the tumor treatment effect is proportional to the depth of stromal cell invasion since TGF synthesis by fibroblasts can cause immune cell efflux or resistance to chemotherapeutic medicines. Therefore, gene expression patterns in tumor tissue can be used to show the link between the tumor microenvironment and patient prognosis. TCGA delivered genomic profiles as well as clinical data, which made it feasible to study the association between genomic features and clinical as well as prognostic aspects. In this study, we observed that GABRD expression was positively associated with the expression of T cells regulatory (Tregs), macrophages M0, while negatively associated with the expression of T cells CD8, T cells follicular helper, macrophages M1, dendritic cells activated, eosinophils, and T cells CD4 memory activated. Based on our findings, it appeared as though GABRD may play a significant part in the TME. In light of these findings, we conducted additional research to investigate the possible connections between GABRD and immunological checkpoints, immunosuppressive genes, chemokines, and chemokine receptors. We found that GABRD expression was positively associated with CD40, TNFSF14, LAIR1, TNFRSF4, TNFRSF18, CD40LG, TNFRSF25, TNFRSF8, NRP1, CD27, and CD276, while negatively associated with HHLA2 and CD44. Based on these findings, GABRD appears to have a tight connection with the regulation of the immune system. Patients with tumors that have high GABRD expression levels could develop an immunosuppressive condition.

We evaluated the connection between GABRD expression and IC50 values of anticancer therapies using data from the GDSC database in order to investigate the possible role that GABRD plays as an indicator in the process of selecting anticancer medications. We found that the IC50 values of BI-2536, bleomycin, embelin, FR-180204, GW843682X, LY317615, NSC-207895, rTRAIL, and VX-11e increased in the high-GABRD group. Based on these findings, it appeared that individuals who had high levels of GABRD expression may be unable to benefit from the therapies provided by the aforementioned medications.

In the end, we must clarify the limitations of this research. Firstly, the sample size is not particularly huge; therefore, it is important to conduct extensive clinical tests. Secondly, we did not assess the expression profiles of GABRD in the serum samples taken from COAD patients. Investigating the serum biomarkers might be an effective way to evaluate treatment responses in real-time. Thirdly, there is a lack of information on the function of GABRD in the control of COAD carcinogenesis on both the cellular and molecular levels.

## 5. Conclusions

Overall, the findings of our research showed that the GABRD expression level in COAD patient tissues was significantly higher than that in normal tissues. In addition to that, GABRD may be a novel prognostic biomarker for COAD patients. The nomogram model can effectively predict patient survival in clinical practice. In addition to this, one of the benefits of our research was that it was the first study to investigate whether or not there is a connection between GABRD expression and TME.

## Data Availability

Some or all data generated or used during the study can be obtained from the corresponding author upon request.
